# Early-life perturbations in glucocorticoid activity impacts on the structure, function and molecular composition of the adult zebrafish (*Danio rerio*) heart

**DOI:** 10.1016/j.mce.2015.07.025

**Published:** 2015-10-15

**Authors:** K.S. Wilson, J. Baily, C.S. Tucker, G. Matrone, S. Vass, C. Moran, K.E. Chapman, J.J. Mullins, C. Kenyon, P.W.F. Hadoke, M.A. Denvir

**Affiliations:** The British Heart Foundation Centre for Cardiovascular Science, University of Edinburgh, The Queen's Medical Research Institute, Edinburgh EH16 4TJ, UK

**Keywords:** Zebrafish, Heart, Glucocorticoids, Development, Echocardiography, Dex, Dexamethasone, *ef1α*, elongation factor 1 alpha, *fgf*, fibroblast growth factor, *gapdh*, glyceraldehyde 3-phosphate dehydrogenase, *gata4*, gata binding factor 4, GC, Glucocorticoid, GCs, Glucocorticoids, GR, glucocorticoid receptor (various, see gene nomenclature below), (GR −/−), glucocorticoid receptor knockdown, GFP, green fluorescent protein, HPA, hypothalamic-pituitary-adrenal, hpf, hours post fertilisation, HPI, hypothalamic-pituitary-interrenal, H&E, hematoxylin and eosin, *igf1*, insulin-like growth factor 1, IOD, inflow: outflow distance, *mef2c*, myosin enhancer factor 2c, Mo, morpholino, *nkx2.5*, homeobox protein Nkx-2.5, PBS, phosphate buffered saline, *pdgf*, platelet derived growth factor, PFA, paraformaldehyde, *plb*, phospholamban, qRT-PCR, quantitative real-time polymerase chain reaction, *ryr*, ryanodine receptor, *serca*, sarco/endoplasmic reticulum Ca2+-ATPase, Tg (CMLC2:GFP), transgenic cardiomyosin light chain 2: green fluorescent protein, UPL, universal probes library, *vmhc*, ventricular myosin heavy chain

## Abstract

**Background:**

Transient early-life perturbations in glucocorticoids (GC) are linked with cardiovascular disease risk in later life. Here the impact of early life manipulations of GC on adult heart structure, function and gene expression were assessed.

**Methods and results:**

Zebrafish embryos were incubated in dexamethasone (Dex) or injected with targeted glucocorticoid receptor (GR) morpholino knockdown (GR Mo) over the first 120 h post fertilisation (hpf); surviving embryos (>90%) were maintained until adulthood under normal conditions. Cardiac function, heart histology and cardiac genes were assessed in embryonic (120 hpf) and adult (120 days post fertilisation (dpf)) hearts.

GR Mo embryos (120 hpf) had smaller hearts with fewer cardiomyocytes, less mature striation pattern, reduced cardiac function and reduced levels of *vmhc* and *igf* mRNA compared with controls. GR Mo adult hearts were smaller with diminished trabecular network pattern, reduced expression of *vmhc* and altered echocardiographic Doppler flow compared to controls. Dex embryos had larger hearts at 120 hpf (Dex 107.2 ± 3.1 vs. controls 90.2 ± 1.1 μm, p < 0.001) with a more mature trabecular network and larger cardiomyocytes (1.62 ± 0.13 cells/μm vs control 2.18 ± 0.13 cells/μm, p < 0.05) and enhanced cardiac performance compared to controls. Adult hearts were larger (1.02 ± 0.07 μg/mg vs controls 0.63 ± 0.06 μg/mg, p = 0.0007), had increased *vmhc* and *gr* mRNA levels.

**Conclusion:**

Perturbations in GR activity during embryonic development results in short and long-term alterations in the heart.

## Highlights

• Embryonic glucocorticoid exposure can have direct impact on cardiac development.
• Altered glucocorticoid activity change the developing heart structure and function.
• Changes in structure, function and gene expression are also observed in later life.
• These subtle changes in the adult heart may increase the risk of disorder or dysfunction.

## 1 Introduction

Epidemiological studies in humans indicate that prenatal glucocorticoid (GC) excess is associated with increased risk of cardio-metabolic disease in later life (Seckl and Meaney, 2004). Rodents exposed to excess GCs during foetal life are more likely to be hypertensive, obese and display impaired glucose tolerance as adults (Nyirenda et al., 1998). While these observations suggest a link between transient changes in early life GCs and cardiovascular risk factors it remains unclear whether this risk also includes changes in the structure and function of the heart itself.

Recent work has supported a link between reduced GC receptor (GR) signalling during early life and altered structure and function of the foetal heart. Global GR null mice (GR−/−), at embryonic day (E) 17.5, have smaller hearts with altered contractility, myofibrillar patterning and expression of calcium handling genes. These mice die soon after birth and so the longer term effects on the adult heart have not been studied. Mice with cardiomyocyte/vascular smooth muscle cell specific disruption of GR (SMGRKO mice) however do survive into adulthood and these have larger hearts with evidence of fibrosis and impaired contractility (Richardson et al., 2013).

Genetic knockout models result in long-term reductions in endogenous GR signalling which, while interesting and important, are unable to address the impact of short term changes in signalling during embryonic development. Models of GC excess typically involve treatment of pregnant rats and sheep with synthetic GC; have addressed the impact of increased exogenous GR signalling during foetal development. These latter models are confounded by GC-induced changes in the maternal hypothalamic-pituitary-adrenal (HPA) axis. They are also unable to address both an increase and a decrease in GR signalling in a contemporaneous experiment using animals with similar genetic background maintained in identical environmental conditions.

The zebrafish circumvents the problems of maternal or placental effects on the developing cardiovascular system, allowing investigation of direct effects of GCs on the developing embryo. In addition, the zebrafish exhibits very similar GC metabolism to mammals, with many key enzymes and pathway products of steroidogenesis similar to those observed in humans (Schoonheim et al., 2010), including the predominance of cortisol as the main circulating corticosteroid. This system, referred to as the hypothalamus-pituitary-interrenal (HPI) axis in teleost fish, is analogous to the mammalian HPA axis (Alsop and Vijayan, 2008). In addition, the zebrafish GR gene (*gr*), of which there is only one version, is remarkably similar in structure to the human form (*GR*) (Aluru and Vijayan, 2009). The zebrafish GR has garnered increasing interest with a number of publications highlighting the importance of this receptor in development (Hillegass et al., 2007; Kondo, 2007; Nesan et al., 2012; Nesan and Vijayan, 2013a; Pikulkaew et al., 2011). However little is known about the effects of transient disruption of GC signalling in early development and even less is known about the long-term effects into adulthood. While multiple methods exist to allow disruption of GC one key technique used in this study is morpholino (Mo) knockdown targeted at the GC receptor (*gr*) gene. This technology has been used extensively by developmental biologists in the zebrafish. However, its use as an approach to create a temporary or short-term knockdown of gene activity, in this case the *gr*, and subsequent long term study of organ structure and function has not been previously reported.

In the work presented here we address the hypothesis that even modest changes in GC activity, either through excessive GC exposure, or reduced GC action, during embryonic development would result in short and long-term effects on the structure and function of the heart. Furthermore, we predicted that these findings would be accompanied by changes in molecular pathways known to influence function and structure of the heart which may in turn impact on its ability to respond to increased work load, stress or injury.

## 2 Methods

### 2.1 Zebrafish husbandry

All animal experimentation was carried out in accordance with the accepted standards of humane animal care under the regulation of the Animal (scientific procedures) Act UK 1986, following experimental approval by the University of Edinburgh animal ethics committee.

Zebrafish transgenic cardiac myosin light chain two: green fluorescent protein (Tg (CMLC2:GFP)) fish were used throughout this study and were housed in 10 L tanks (3 fish/L) and maintained under standard conditions (14:10 h light: dark photoperiod, at ambient temperature of 28.5 °C (Kimmel et al., 1995). Eggs were collected and stored in “systems water” comprising 6 g salt (Tropic Marin^®^ Wartenberg, Germany) and 0.5 mg/L of the antiseptic methylthioninium chloride (methylene blue) in 20 L of in-house deionised H_2_O. All fertilised age-appropriate eggs (2 cell stage (∼1 h post fertilisation (hpf)) (Kimmel et al., 1995) were either injected with Mo (or control) or treated with drug (or vehicle) and housed in 10 cm petri dishes at 28.5 °C. Batches of embryos that had undergone GC manipulation during the first 120 hpf were allowed to grow until adulthood (120 days post fertilisation -dpf) under normal husbandry conditions. These fish were closely monitored for distress or structural abnormalities throughout their growth and showed no significant excess of morbidity or mortality compared to controls.

### 2.2 Embryonic GR manipulation

Mo were obtained from GeneTools (Philomath, Oregon, USA) to down-regulate the zebrafish GR gene (*gr*). As the primary aim of this study was to determine the long-term effects of transient knock down in Gr protein in the zebrafish heart a start site targeted Mo (ATG Mo) was designed, this Mo is targeted to the 5′ untranslated region (5′ UTR) of the mRNA encoding Gr protein (ATG Mo- cattctccagtcctccttgatccat) to prevent the transcription of the Gr protein. The use of an ATG Mo is a convenient means of knocking down expression of the protein and learning how that knockdown changes the cells or organism. As knockdown with and ATG Mo prevents the translation of the coding region of the targeted transcript confirmation of ATG Mo success was carried out by western blot analysis of the total Gr protein (see western blot analysis). As a control for the ATG Mo a 5 bp mispair Mo was designed (Mism Mo- cattgtccactcctgcttcatcgat). Each Mo incorporated a red emitting fluorescent tag to confirm integration throughout the developing embryo. Microinjection of Mo was performed at the 2-cell stage using a standard approach (Nusslein-Volhard and Dahm, 2002) with a IM-300 Narishige programmable micro-injector. Volume of Mo injected was calculated by measuring the droplet radius (bolus volume = 4/3 πr^3^). Careful titration studies were performed to select a concentration of Mo which avoided off-target, non-specific developmental abnormalities while also producing consistent modest knockdown of Gr protein levels, the concentration selected for was 3 ng/nL. The 120 hpf survival of this group 78.25 ± 1.11% vs Mism Mo 84.00 ± 1.47%, p = 0.02.

To create excess exogenous GR signalling during early life, embryos (between 24 and 120 hpf) were exposed to the synthetic GR agonist dexamethasone (Dex) in the bathing water. This was dissolved in ethanol and added to the systems water from 24 to 120 hpf. Systems water containing vehicle [0.1% total volume ethanol] was included as experimental control. Solutions were replaced every 48 h. A Dex dose-range study (1–200 μM) was undertaken as part of a series of separate experiments to establish an optimal concentration of 100 μM (Wilson et al., 2013). Briefly, the Dex concentration range was selected based on published pharmacological data, while the upper limit may appear relatively high in comparison to mammalian studies, these concentrations were selected based on previous zebrafish publications (Hillegass et al., 2007; Schaaf et al., 2008). Furthermore the selected concentration was chosen as it significantly altered physiology (Wilson et al., 2013) but did not impact on survival (Dex 87.33 ± 0.88% vs vehicle only controls 93.00 ± 2.08%, p = 0.06) or gross morphology (Wilson et al., 2013).

### 2.3 Embryo to adult growth

Batches of embryos that had undergone GC manipulation as outlined above, with either GR Mo (ATG Mo) or Dex, and displayed no significant structural or phenotypic abnormalities as recommended by Home Office veterinary officers, were allowed to grow until adulthood (120 dpf) under normal husbandry conditions (as detailed in husbandry section). These adult fish were closely monitored for distress or structural abnormalities throughout their growth until 120 days.

### 2.4 Functional assessment of embryonic hearts

The functional impact of GR modulation was assessed using Tg (CMLC2: GFP) zebrafish embryo hearts visualised using an Axioskop II MOT compound microscope with a (40×) dipper objective lens. Videos and still frame images were captured using a black and white camera and analysis was carried out via attached PC. Ventricle length, width, area and volume were assessed in diastole and systole for three cardiac cycles using image analysis software (ImageJ). Ventricle area was measured assuming the ventricle was elliptical in shape and using the equation (area = *π* *ab* (where *a* is half the long axis length and *b* is half the short axis length)) were assessed in diastole and systole for three cardiac cycles using image analysis software (ImageJ). Heart rate was assessed from these captured images over 3 cardiac cycles using frame rate (30/second) as the unit of time. Stroke volume was assessed by measuring ventricle diastolic and systolic area and then assuming the ventricle was ellipse shape. The volume change during the cardiac cycle was estimated from the formula volume = 4/3 *π* *abc* (where *a* and *b* are described above and *c* is half the depth in the z-plain). Cardiac output was estimated as the product of the stroke volume and heart rate for each heart. Ejection fraction was estimated as percentage area change between diastole and systole using the formula (diastolic area-systolic area) ÷ diastolic area, expressed as a percentage.

### 2.5 Echocardiography of adult hearts

Adult heart functional assessment was carried out by ultrasound (VisualSonics, Vevo 770). Zebrafish were anaesthetised using MS222 and placed on their dorsal surface in a shallow bath containing induction strength MS222 (4.2%) and gently restrained using a Plastiscine mould. A RMV 711, central frequency 55 MHz transducer was used to acquire B-mode and spectral Doppler images in transverse and longitudinal planes of the ventricular inflow and outflow signals. Images were captured representing 5–10 typical cardiac cycles and analysed by a single experienced operator (AT). B-mode images were analysed for area change of the ventricle during the cardiac cycle in the long axis view and Doppler signals were analysed for peak in-flow and out-flow velocities and for associated acceleration and deceleration values and times.

### 2.6 Embryonic heart histology

Heart ventricles from embryos (120 hpf) were isolated and stained with DAPI (nuclear stain). These were then mounted in ProLong antifade mounting agent (Life technologies) and detailed images obtained using a confocal microscope. Cardiomyocytes were counted in each ventricle using a previously validated technique (Matrone et al., 2013) from Z-stacks in 3 μm sections moving progressively through the ventricle counting nuclei which clearly overlapped with GFP signal.

### 2.7 Adult heart histology

Histology was performed on adult hearts using a protocol previously published (Sabaliauskas et al., 2006). Briefly, isolated hearts were fixed in 4% paraformaldehyde (PFA) overnight then washed 3 times with 1 X phosphate buffered saline (PBS). Hearts were stored in methanol until embedding in 2% agarose which were subsequently paraffin embedded. These paraffin blocks were then microtome sectioned into 5 μM thin sections onto microscope slides. After drying sections were stained with haematoxylin and eosin (H&E) staining was performed using a standard protocol (Fischer et al., 2008). Ventricle Cavity Area was assessed quantitatively using ImageJ on H&E stained sections, where ventricle size was calculated by tracing around the outer ventricular wall using the free-hand tool, inter-trabecular space was then determined by tracing around areas of free-space (unstained with no apparent tissue) within this ventricle. The regions of empty space were then totalled to give the free area within the section. This inter-trabecular space was then subtracted from the total ventricular area and the total percentage of inter-trabecular was thus given as a percentage. This was carried out on a minimum of 6 × 5 μM sections per heart and for 8 hearts from each treatment group.

### 2.8 Heart isolation for RNA extraction

Embryonic hearts were isolated using a previously published method utilising mechanical agitation(Burns and MacRae, 2006). Briefly, embryos were pooled in a 1.5 ml Eppendorf tube and a 19 gauge needle with regular bevel attached to a 5 ml syringe was inserted and repeatedly plunged (30–40 times) to homogenise the embryos. The resulting homogenate was filtered through a 105 μm nylon mesh, the perfusate was collected and GFP positive hearts were selected under fluorescent microscope. Isolated hearts were stored in RNAlater for qRT-PCR. Adult fish were culled by anaesthetic overdose (MS-222) and hearts removed by surgical dissection and placed in a small volume of RNAlater solution.

#### 2.8.1 RNA extraction and qRT-PCR

Total RNA was extracted from pools of isolated embryonic hearts (120 hpf, 150 hearts per sample) or pooled adult hearts (5 hearts per sample) after initial homogenisation using a bead mixer mill (3 min 15 Hz) using the RNeasy Mini kit (Qiagen) according to manufactures instructions. Following this, DNA-free™ kit (Applied Biosystems, Warrington, UK) was used to eliminate possible gDNA presence. RNA quantity was determined using a Nanodrop^®^ spectrophotometer ND-1000 (Fisher Scientific, Loughborough, UK) to determine extracted RNA concentration at 260 nm (extinction coefficient 40 ng/μL). RNA integrity was verified by a 260/280 nm absorbance ratio of ∼2 and 1% agarose gel electrophoresis.

Total RNA was reverse transcribed using the High capacity cDNA Reverse Transcription Kit (Applied Biosystems, Warrington, UK) according to manufacturer's guidelines. qRT-PCR was performed to quantify the expression of a number of gene mRNA (for sequences see supplemental Table 1) using the LightCycler 480 system (Roche, Hertfordshire, UK) with universal probes library (UPL) probes (Roche Diagnostics Ltd, UK) at the standard operating conditions 95 °C for 5 min, 50 cycles (95 °C 10 s, 60 °C 30 s, 72 °C 1 s), 40 °C 30 s). For each experiment an appropriate housekeeping gene was run concurrently which enabled gene of interest quantification using the LightCycler software, data produced is through maximum second derivative method, whereby the entire amplification curve is considered rather than just the threshold point.

### 2.9 Protein extraction and analysis

Protein was obtained from pooled embryonic (150 hearts) or adult hearts (10 hearts) or pooled whole embryos (10 embryos) by a standard protocol. Pooled hearts were covered in radioimmunoprecipitation assay (RIPA) buffer 50 μL (containing phenylmethylsulfonyl fluoride (1 mM) and protease inhibitors [1:100] (Sigma–Aldrich protease inhibitor cocktail P2714: AEBSF 2 mM, E−64 14 μM, Bestatin 130 μM, Leupeptin 0.9 μM, Aprotinin 0.3 μM, EDTA 1 mM) and homogenised in a Soniprep Ultrasonic Homogenizer (Sonciator – Model 3000 MP Ultrasonic Homogenizer, BioLogics). Samples were run as per manufacturer's instructions, with SDS Bis-Tris buffer at 100 V for 45 min. The gel was then transferred to nitrocellulose membrane by a semi-dry transfer method (200 mA for 120 min) using transfer buffer (Tris base, pH 8.3, 25 mM, glycine 192 mM, methanol 20%). Membranes were blocked and probed following a standard western blotting technique. Primary antibody used in this work was a GR antibody (1:100 dilution of a goat anti-trout GR) which was a kind gift from Dr M Vijayan, University of Waterloo, Canada (Pikulkaew et al., 2011).The secondary used was a rabbit anti goat antibody conjugated to horse radish peroxidase (HRP) (1:1000) (Santa Cruz Biotechnology, Heidelberg, Germany, Cat no sc-2768) allowing detection by enhanced chemiluminescence (ECL) reagent (GE Healthcare, Buckinghamshire, UK).

### 2.10 GR morpholino rescue

To determine whether the effects observed for the GR Mo were due specifically to Gr knock-down or due to non-specific effects a rescue experiment was carried out using a modified 5′-UTR zebrafish *gr* mRNA (gr-RNA). As the rescue has a modified 5′UTR it contains no target for the ATG Mo but the coding region of the rescue contains the sequence to encode the protein of interest. Template DNA was obtained in the form of IMAGE clone (Source Bioscience, Cambridge), in purified plasmid Miniprep form. The *gr* gene was cloned into the pNR-LIB clone (Source Bioscience, Cambridge) and linearized using XhoI restriction enzyme (Applied Biosystems). Capped transcription reaction was carried out using the mMessage mMachine Kit (Ambion) according to manufacturer's instruction. Final capped mRNA concentration was achieved by dilution in sterile RNAse free dH_2_0.

### 2.11 Statistical analysis

Experiments were performed triplicate with on average 20–30 larvae per experiment, unless otherwise stated. Data are mostly presented as mean ± standard error of the mean (SEM) unless otherwise stated. Statistical analyses were performed using GraphPad Prism 5. One-way or two-way repeated measures analysis of variance (ANOVA) followed by Bonferroni or Dunnett's post-hoc tests were used to compare means within and between groups. P values < 0.05 were considered significant.

Data for control groups are presented in most figures as a combined mean value following detailed analysis comparing the control groups, there was no significant difference between the control-Dex and control-Mo embryo data for a wide range of measured parameters (Supplemental Table 1). Statistical differences stated for the treatment groups however were between treatment and their specific control group.

## 3 Results

### 3.1 Glucocorticoid manipulation impacts on structure and function of the embryonic heart

**Fig. 1:**
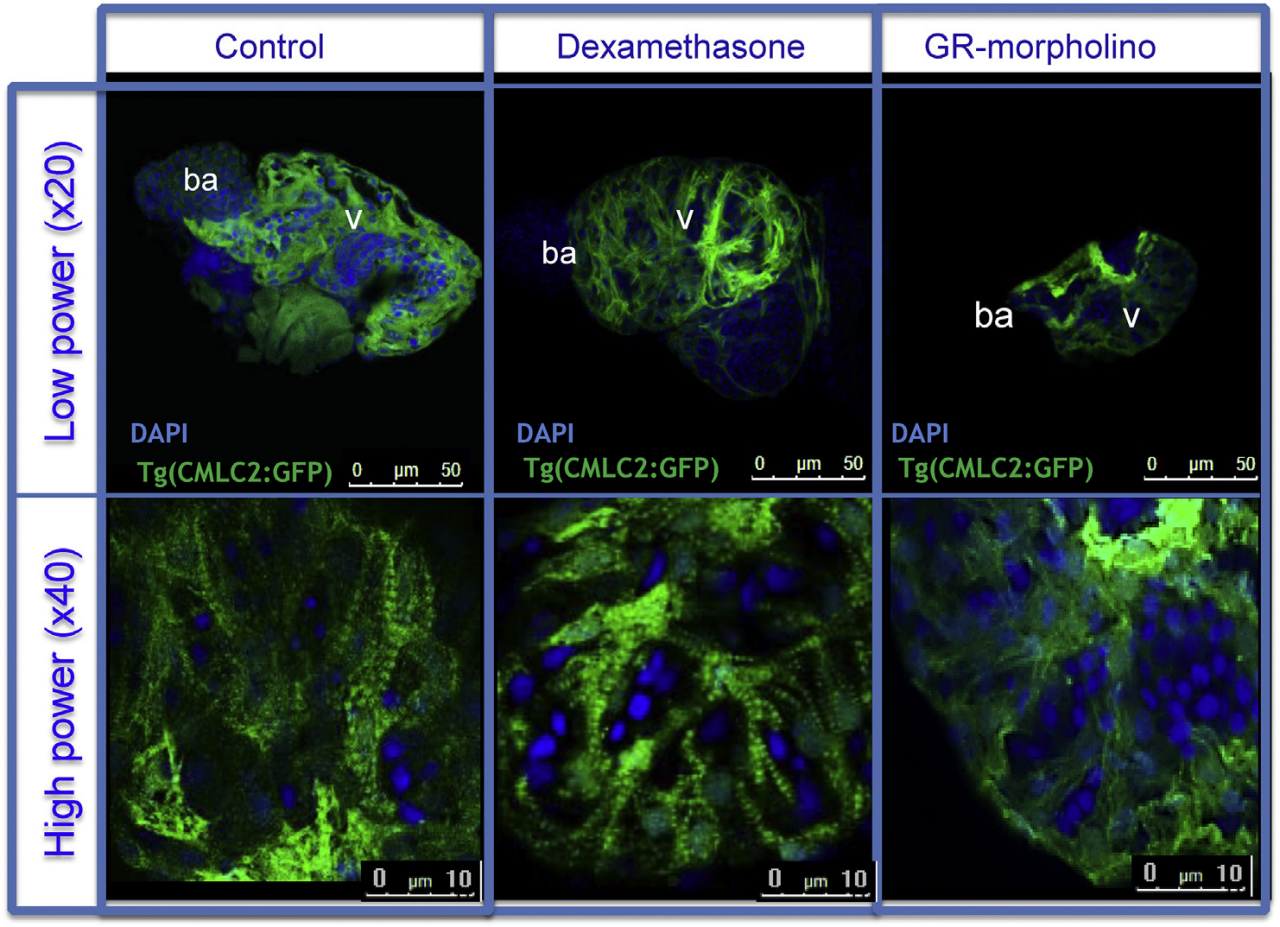
Manipulation of glucocorticoid activity induces changes in cardiac structure Confocal images of embryonic (120 h post fertilization (hpf)) of Tg (CMLC2: GFP) zebrafish hearts after 120 h glucocorticoid receptor (GR) manipulation with either GR agonist dexamethasone, (Dex) [100 μM] or targeted GR knockdown using morpholino (GR Mo). Whole mounted GFP embryonic hearts (green) were stained with DAPI (blue) and imaged at high power with a confocal microscope. Gross cardiac morphology can be observed at ×20 magnification, higher power images (×40 magnification) demonstrate advanced maturity of the Dex treated hearts in contrast to the GR Mo hearts which show considerable immaturity of myofibrillar and trabecular patterning. (For interpretation of the references to colour in this figure legend, the reader is referred to the web version of this article.)

In contrast to control (age-matched and untreated) hearts at 120 hpf, which displayed a clear striation pattern with a mature trabecular network (Fig. 1), hearts from GR Mo embryos were smaller with a less well developed trabecular network and absent striations. Dex treated hearts, in contrast to the GR Mo hearts, showed a mature trabecular network with well-developed striation patterns.

**Fig. 2:**
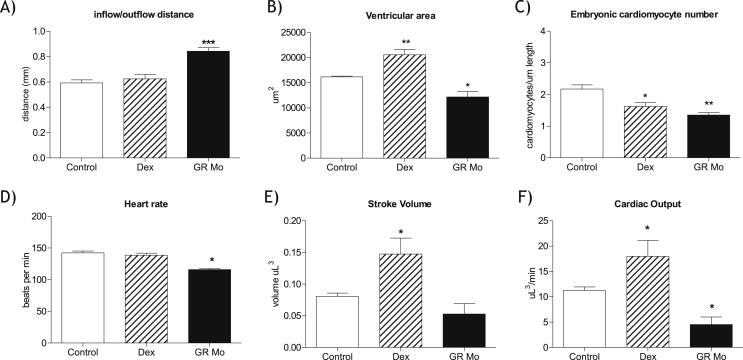
Effects of glucocorticoid manipulation on development, structure and function of the embryonic heart Structural and functional observations of embryonic hearts were carried over 120 h post fertilization (hpf) using Tg (CMLC2:GFP) zebrafish which had been exposed to the glucocorticoid receptor (GR) agonist dexamethasone (Dex) [100 μM] for 120 h or treated with targeted GR translational blocking morpholino (GR Mo). Results were compared with controls which are a mean of Dex (vehicle only) controls and GR Mo (mism Mo) controls. Parameters investigated were A) Inflow/outflow area (measured at 72 hpf), an indicator of cardiac looping from cardiac tube to two chambered heart, B) ventricular area, a marker of cardiac growth and development, C) cardiomyocyte number normalized to heart length, D) heart rate, E) stroke volume and F) cardiac output. Data are mean ± SEM and were compared to control by one-way ANOVA and Dunnett's post hoc test. A) inflow/outflow distance n = (20 ***p ≤ 0.0001, B) ventricle area n = 4 experiments, (6 hearts per experiment) ± SEM *P ≤ 0.05, **P ≤ 0.001, C) ventricle cardiomyocyte number (normalized to body length) n = 12 hearts *p ≤ 0.05, **p ≤ 0.01. D–F), all cardiac function assessments n = 4 experiments (12 embryos per experiment) *p ≤ 0.05.

Ventricle inflow: outflow distance (IOD), a measure of the degree of cardiac looping during development, was increased at 72 hpf in GR Mo treated embryos suggesting delayed heart development (Fig. 2A). Ventricles from GR Mo treated embryos showed reduced cross-sectional area compared to controls (Fig. 2B) and reduced number of ventricular cardiomyocytes per unit heart volume (Fig. 2C). Heart rate and cardiac output were reduced in GR Mo embryos (Fig. 2D and F). In Dex-treated embryos, ventricle inflow: outflow distance was not different from controls (Fig. 2A), cross-sectional area was increased (Fig. 2B) and number of cardiomyocytes per unit heart volume was reduced compared to controls (Fig. 2C). Dex-treated embryos had similar heart rates but greater stroke volume and greater cardiac output compared to controls (Fig. 2D–F).

**Fig. 3:**
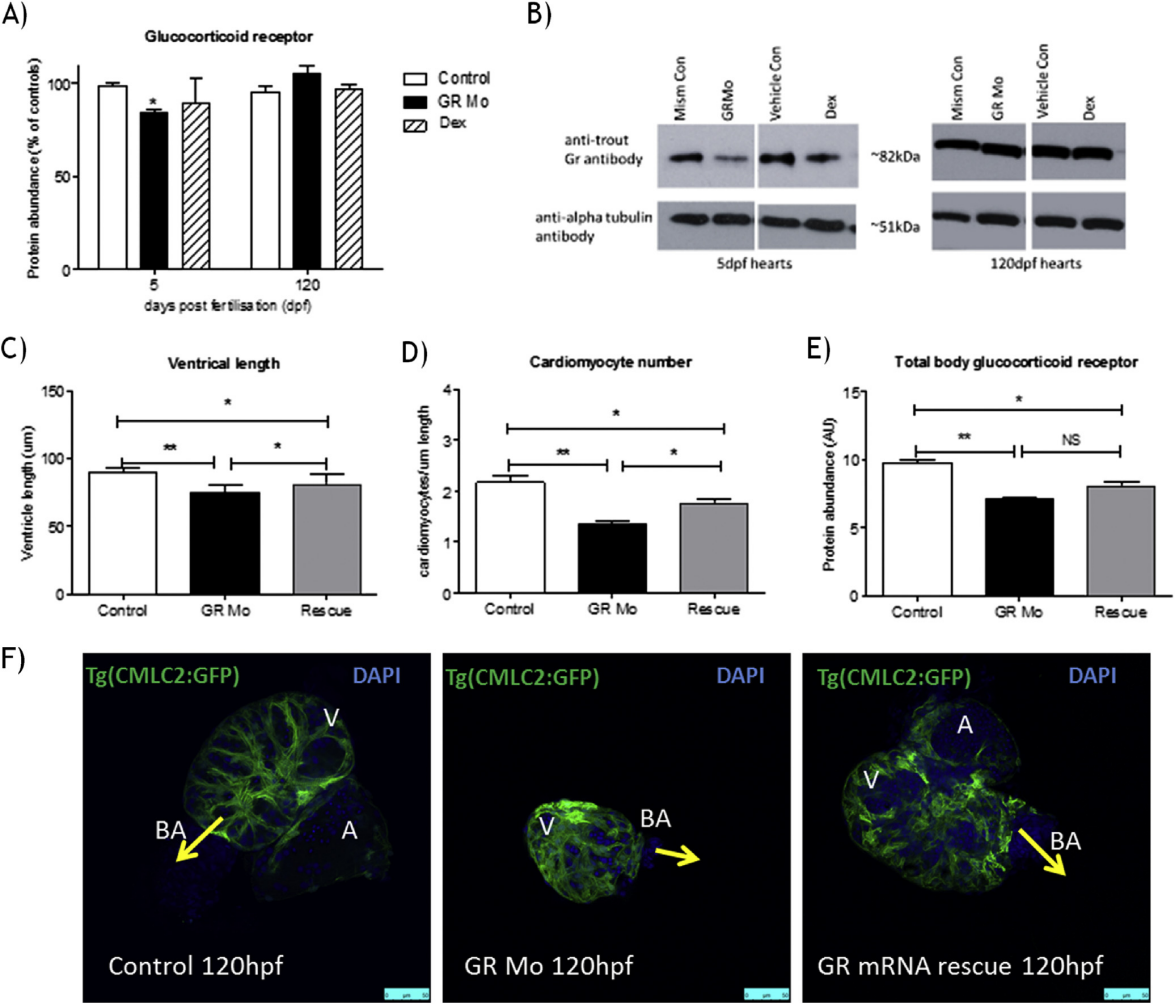
Effects of glucocorticoid receptor morpholino and rescue on the embryonic heart Western blot analysis was carried out to determine whether glucocorticoid receptor protein (Gr) was reduced in isolated hearts from zebrafish embryos (5 days post fertilization (5 dpf)) or adults (120 dpf) which had been treated with either glucocorticoid receptor (GR) translational blocking morpholino (GR Mo) or with GR agonist dexamethasone (Dex) [100 μM] for 120 h (5 dpf). A) Densitometry of Western blots of Gr abundance in isolated hearts. Data are n = 3 (150 embryo heart or 10 adult hearts per n), given as a mean percentage of their respective controls (mism Mo for GR Mo and vehicle for Dex) ± SEM analysed by two-way ANOVA and Bonferroni post hoc test. *p ≤ 0.05. B) Example of Western blot of embryonic (5 dfp) and adult (120 dpf) heart tissue lysates probed with antibodies against Gr with alpha-tubulin (loading control), C) Success of GR Mo was further determined following co-injection of rescue mRNA with GR Mo and calculation of ventricle length at 120 h post fertilization (hpf) D) Calculation of cardiomyocyte number at 120 hpf. C and D) n = 3 (6 embryos per group). Data are mean ± SEM analysed by two-way ANOVA and Bonferroni post hoc test vs each individual group. *p ≤ 0.05 and **p ≤ 0.01. E) Densitometry of Western blots of Gr abundance in 120 hpf whole embryo homogenate. Data are n = 3 (10 embryo per n), data given as relative protein abundance for control (mism Mo), GR Mo, and Gr mRNA rescue (rescue) n = 3 mean ± SEM analysed by two-way ANOVA and Bonferroni post hoc test, *p ≤ 0.05, **p ≤ 0.01. F) Confocal images of isolated 120 hpf Tg (CMLC2: GFP) zebrafish hearts (green) co-stained with DAPI nuclear stain (blue), images are isolated from embryos which were treated with control morpholino (mism Mo), GR Mo and capped GR mRNA (rescue) co-injected with GR Mo. (For interpretation of the references to colour in this figure legend, the reader is referred to the web version of this article.)

Isolated hearts from GR Mo embryos (120 hpf) were confirmed to have lower Gr protein abundance than controls, (18.60 ± 4.45% reduction compared to age matched controls p < 0.05. By adulthood however (120 dpf) there was no difference in heart Gr protein in GR Mo adults compared to controls confirming the transient nature of this genetically induced reduction in GR activity (Fig. 3A and B). In contrast hearts isolated from embryos treated with Dex showed no significant alteration in Gr abundance compared to controls at either 120 hpf or 120 dpf (Fig. 3A and B).The effects of GR Mo on heart size, cardiomyocyte number and total embryo protein expression were partially rescued by co-treatment with a *gr*-RNA (rescue), with heart size and cardiomyocyte number both greater than GR Mo alone, but still reduced compared to mism controls (Fig. 3C and D). In contrast to control isolated hearts (120 hpf), a lack of clear striation pattern was observed in GR Mo embryo hearts, however striation was observed in hearts isolated from embryos co-treated with GR Mo and *gr*-RNA (Fig. 3E).

### 3.2 Glucocorticoid manipulation impacts on gene expression in the larval heart

**Fig. 4:**
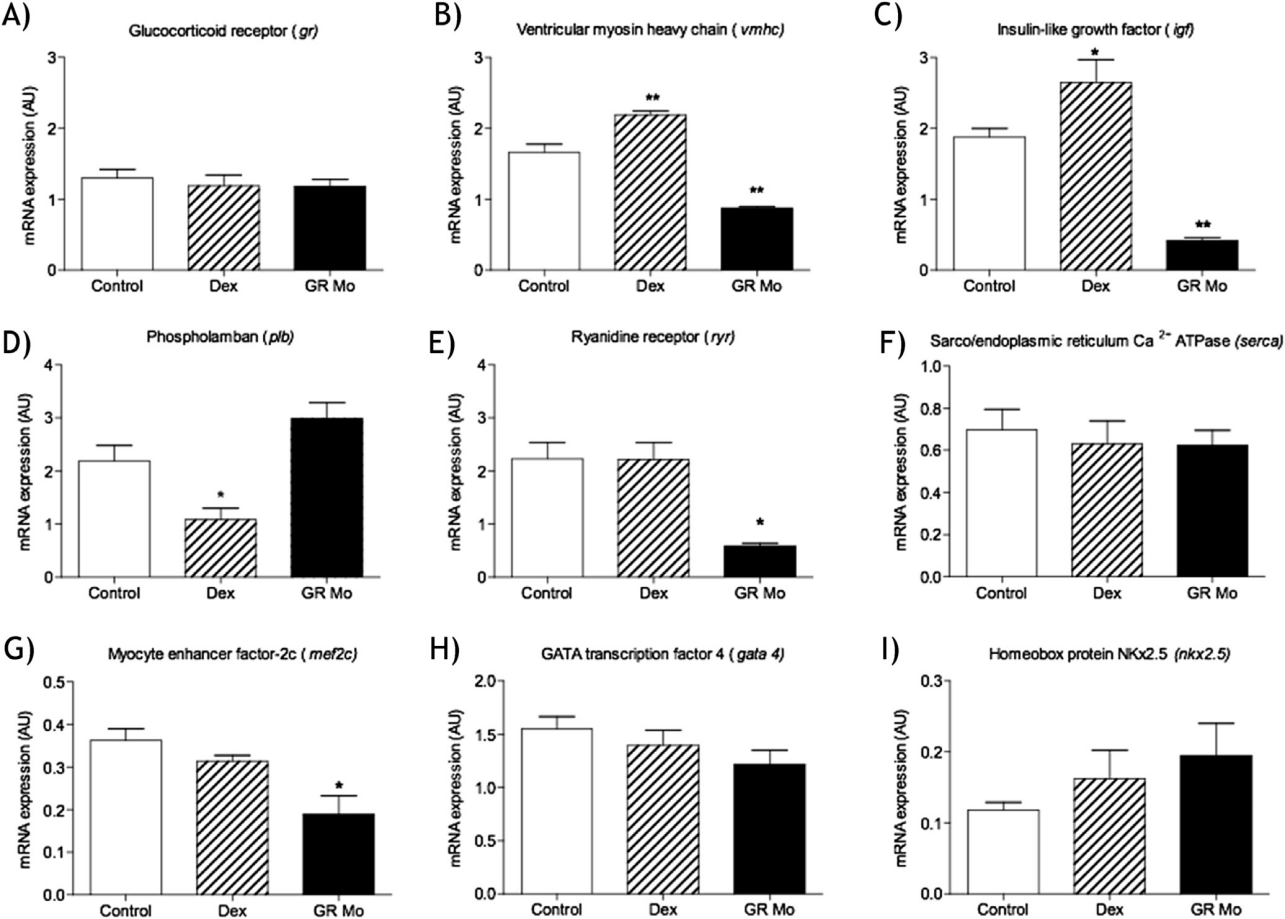
Effects of glucocorticoid manipulation on embryonic heart genes Relative mRNA abundance was determined in isolated hearts at 120 h post fertilization (hpf) after 120 h glucocorticoid receptor (GR) manipulation with either dexamethasone (Dex) [100 μM] or targeted gr morpholino (GR Mo). Genes which were investigated were (A) Glucocorticoid receptor (*gr*) (B) Insulin like growth factor (*igf*) (C) ventricular myosin heavy chain (*vmhc*) (D) phospholamban (*plb*) (E) Ryanadine receptor (*ryr*) (F) sarco-endoplasmic reticulum Ca2+ ATPase (*serca*) (G) myocyte enhancer factor 2 c (*mef2c*) (H) GATA transcription factor 4 (*gata4*) and (I) Homeobox protein NKX2.5 (*nkx2.5*). mRNA expression was determined from isolated hearts at 120 hpf normalized to house-keeping gene, data are mean of n = 3 (150 hearts per n) ± SEM *p ≤ 0.05, **p ≤ 0.01. All data were compared to their controls by one-way ANOVA and Dunnett's post hoc test.

Genes associated with growth and maturity (*igf*, *mef2c* and *vmhc*) were affected by GR manipulation in isolated embryonic (120 hpf) hearts (Fig. 4). No alteration in expression was observed for *gr* mRNA in either the Dex or GR Mo groups (Fig. 4A). Isolated hearts from GR Mo embryos however displayed reduced expression of *vmhc* and *igf* mRNA; in contrast Dex-treated embryos showed increased levels of *vmhc* and *igf* compared to controls (Fig. 4B and C). Phospholamban (*plb*) mRNA expression was found to be decreased in hearts isolated from Dex-treated embryos compared to their controls (Fig. 4D) however no change was noted in these hearts for other genes associated with calcium handling (sarco-endoplasmic reticulum calcium transport ATPase (*serca*) the ryanodine release channel gene (*ryr*) (Fig. 4E and F). While the *ryr* expression was lower in GR Mo embryos there was no difference in other genes associated with calcium handling proteins (Fig. 4D–F). *mef2c* expression was found to be lower in hearts isolated from GR Mo compared to controls, Dex-treatment had no impact on the expression of this gene (Fig. 4G). Neither GR Mo nor Dex treatment were found to alter the expression of *gata4* or *nkx2.5* (Fig. 4H and I).

### 3.3 Transient early-life glucocorticoid manipulation impacts on structure, function and gene expression in the adult heart

**Fig. 5:**
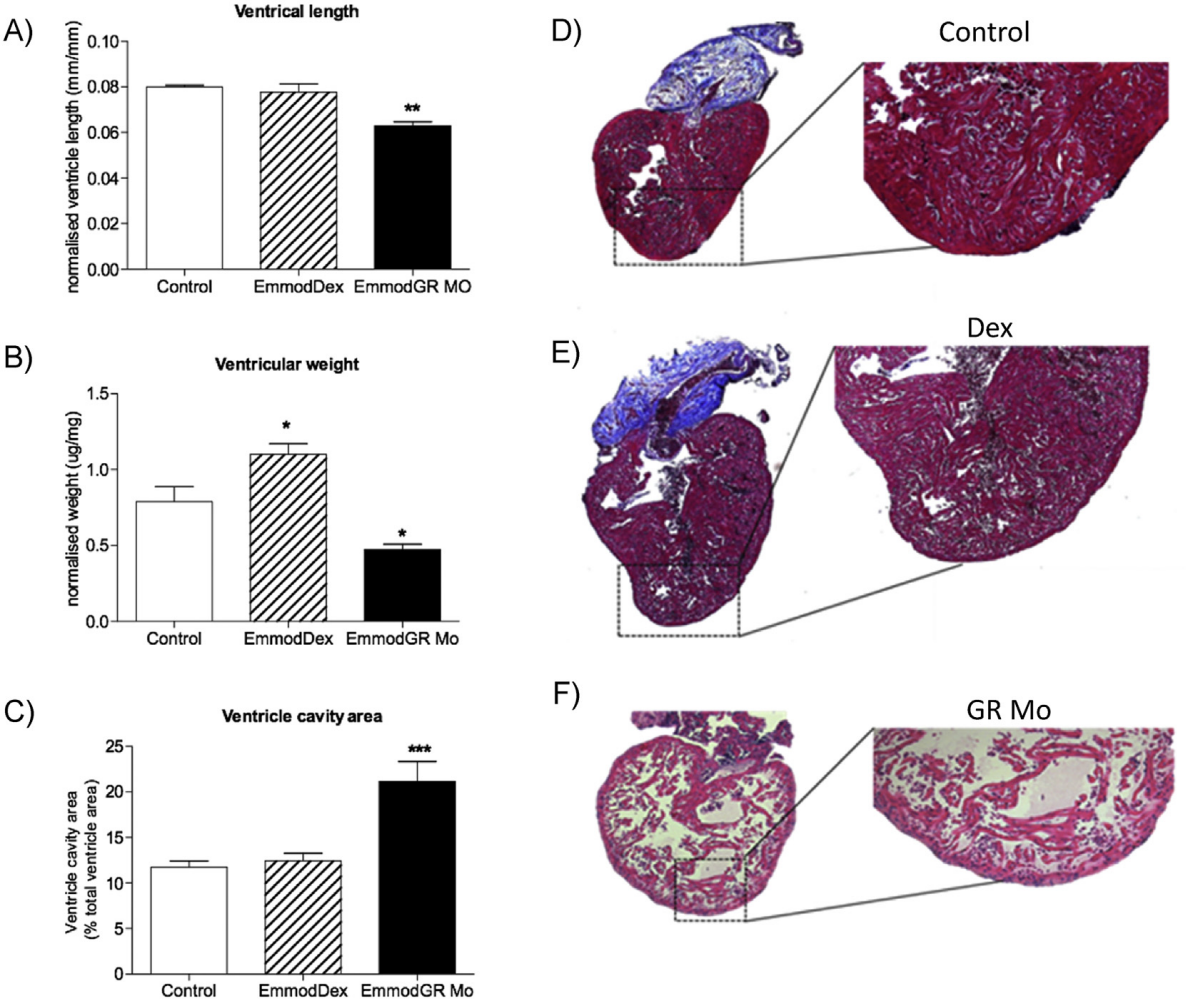
Effects of embryonic glucocorticoid manipulation on adult heart structure Long-term impact of embryonic glucocorticoid receptor (GR) manipulation with either GR agonist dexamethasone (Dex) [100 μM], or targeted translational GR knockdown using morpholino (GR Mo) was assessed in the heart of 120 day post fertilisation (dpf) adult zebrafish. Structural analyses of A) ventricle length, B) weight and C) cavity area were carried out. Data are n = 8 hearts per group, displayed as mean ± SEM analysed by one-way ANOVA with Dunnett's post hoc comparison (*p < 0.05, **p < 0.01, ***p < 0.001). D–F) Histology (haematoxylin and eosin staining) in adult fish hearts D) Controls (Vehicle only control shown here), E) Dex, F) GR Mo.

Adult zebrafish hearts (Fig. 5) derived from GR Mo embryos were shorter in length (normalised to total fish length (Fig. 5A) and weighed less (normalised to body weight (Fig. 5B) compared to controls. Histological examination highlighted these hearts had a more sparse trabecular network (Fig. 5F compared to controls Fig. 5D) which when quantified resulted in an increased ventricle cavity area (Fig. 5C). Adult hearts derived from Dex-treated embryos were heavier with similar length and trabecular pattern compared to controls (Figures A–C); histologically they were similar to controls (Fig. 5D and E).

**Fig. 6:**
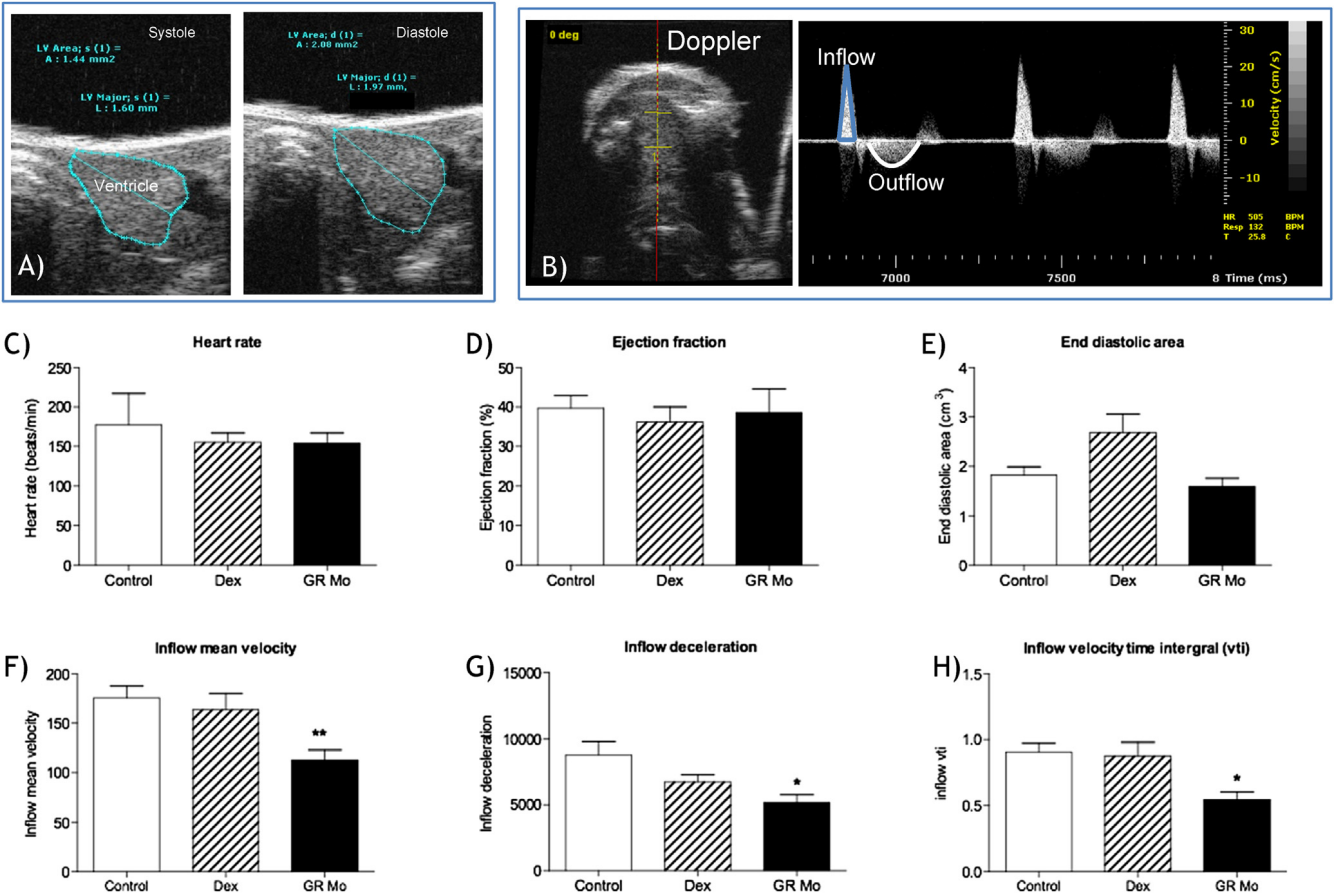
Effects of embryonic glucocorticoid receptor manipulation on adult cardiac function Echocardiography was used to assess function of the adult ventricle using standard B-mode techniques and inflow and outflow Doppler patterns of the ventricle. A) Show typical examples of B-mode images used for calculation of ejection fraction using the epicardial outline in systole and diastole. B) Doppler signals obtained in the transverse plain were used to capture inflow and outflow velocity envelopes. Analysis of all images was undertaken by an independent operator blinded to treatment of the zebrafish. From echocardiography a number of cardiac functional parameters were determined following manipulation of glucocorticoid receptor (GR) such as C) heart rate, D) ejection fraction, E) end diastolic area, F) inflow mean velocity, G) inflow deceleration and H) inflow velocity time integral (vti). Data presented are for adults treated with 100 μM dexamethasone (Dex) as embryos n = 10, those treated with morpholino targeted towards Gr (GR Mo) n = 10, and Controls n = 17 (consisting of Dex controls n = 9 and GR Mo controls n = 8) and displayed as mean ± SEM. Data were analysed by one-way ANOVA and Dunnett's post hoc test, (*p < 0.05 and **p < 0.01).

Echocardiography (Fig. 6) indicated that adult hearts derived from GR Mo embryos had similar heart rate (Fig. 6C), ejection fraction (Fig. 6D) and end diastolic area (Fig. 6E) however had reduced ventricular inflow velocity (Fig. 6F), inflow deceleration time (Fig. 6G) and velocity time integral (Fig. 6H) compared to controls. Dex-treated adult hearts had similar ejection fraction and Doppler signals compared to controls (Fig. 6B–G).

**Fig. 7:**
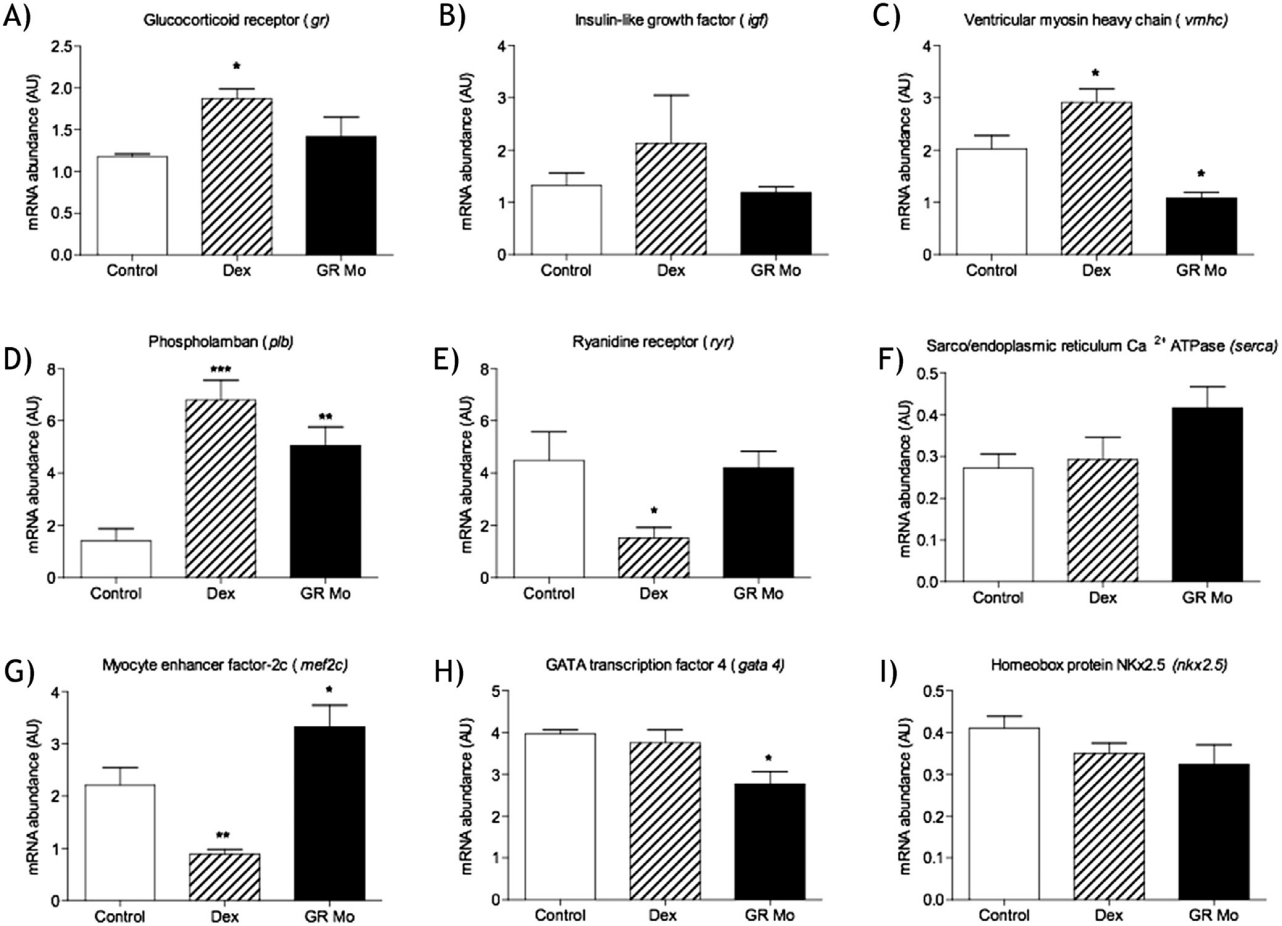
Effects of embryonic glucocorticoid receptor manipulation on adult heart gene expression mRNA expression in adult isolated hearts after glucocorticoid receptor (GR) manipulation with either GR agonist dexamethasone (Dex) [100 μM], or targeted GR knockdown using morpholino (GR Mo). Genes of interest were (A) Glucocorticoid receptor (*gr*) (B) Insulin like growth factor (*igf*), (C) ventricular myosin heavy chain (*vmhc*), (D) phospholamban (*plb*), (E) Ryanodine receptor (*ryr*), (F) sarco-endoplasmic reticulum Ca2+ ATPase (*serca*), (G) myocyte enhancer factor 2 c (*mef2c*), (H) GATA transcription factor 4 (*gata4*) and (I) Homeobox protein NKX2.5 (*nkx2.5*). Relative mRNA expression was determined from isolated hearts at 120 dpf, mean of n = 3 (5 hearts per n) ± SEM, all data analysed compared to control by one-way ANOVA and Dunnett's post hoc test (*p ≤ 0.05, **p ≤ 0.01, ***p ≤ 0.001).

Ventricles from adults treated with Dex during embryonic development had increased expression of *gr*, *vmhc* and *plb* mRNA compared to controls (Fig. 7A, C and D respectively) but displayed reduced *mef2c* and *ryr* mRNA relative abundance compared to controls (Fig. 7I and G respectively). In adult ventricles derived from GR Mo embryos, mRNA levels of *vmhc* (Fig. 7C) and *gata4* (Fig. 7H) were reduced compared to controls while *plb* and *mef2c* mRNA abundance was increased (Fig. 7D and G respectively). No alteration in gene abundance was observed for *igf* and *nkx2.5* in either GR Mo or Dex treated embryos (Fig. 7B and I).

## 4 Discussion

Polymorphisms in GC receptors have been well described in the literature and these have been associated with variations in human response to stress, injury and disease (Quax et al., 2013). The relevance of the study reported here is therefore clear, subtle and transient increases or decreases in GR activity during the life cycle of an organism, particularly during embryogenesis and early development, can have significant long-term effects on the structure and function of a number of GR-sensitive organs and systems including the heart.

Our study has clearly shown that changes in GR signalling during early life can cause long-term alterations in the structure, function and gene expression of the adult heart. The approach of studying both increased and decreased activity of GR in the same model contemporaneously has allowed us to directly compare and contrast the actions of GCs on the embryonic and adult heart.

### 4.1 Diminished GR activity during embryogenesis delays cardiac development and leads to altered cardiac structure and function in adult life

Our findings, using GR Mo knockdown in the teleost fish, are consistent with those described in the (GR −/−) foetal mouse (Rog-Zielinska et al., 2012) where hearts were smaller, with reduced cardiomyocyte size and greater cardiomyocyte density (Richardson et al., 2013). *In-utero* echocardiography of these foetal hearts showed altered Doppler signals suggestive of impaired cardiac function although, unlike the GR Mo zebrafish embryos, they had normal ejection fraction. Similar to the mouse (GR−/−), GR Mo zebrafish embryos (at 120 hpf) displayed changes in expression of key cardiac genes. Alterations in cardiac developmental genes in whole GR Mo treated embryos has been reported previously, using microarray techniques (Nesan and Vijayan, 2013b), The findings reported here are concordant with this study although we have further refined the approach by demonstrating gene changes in isolated embryonic hearts. Together however, these studies underscore the critical role of GC signalling in programming molecular events essential for zebrafish heart development. In particular the observed changes in the mRNA of endoplasmic calcium release channel, found exclusively in developing heart pre-cardiac mesoderm from 14 hpf, is known to play a dual role in contractile function and cardiac development (Wu et al., 2011). Its reduced expression in our GR Mo embryos may therefore account for changes in both development and cardiac contractility in embryonic hearts. Reduced cardiac function in these embryos could also be partly due to a loss of the direct inotropic effects of GCs mediated by a reduction in GR. A direct inotropic effect of GCs has been described in rodent models (Richardson et al., 2013) but not previously in the embryonic zebrafish heart.

One key difference between the experiments described here and the studies in the GR−/− mouse is the transient nature of the Mo knockdown and the subsequent survival of the majority of embryos into adult life. Survival to 120 hpf following GR Mo treatment was similar in this study to previous studies using this (Pikulkaew et al., 2011), and similar to studies treating embryos with glucocorticoids (Nesan et al., 2012). A high level of survival of apparently healthy embryos to the 5 day time point was important in our study for three reasons; firstly, we did not want to produce large numbers of embryos with developmental or structural abnormalities which would then limit their chances of survival to adulthood. This had the potential to create survival-bias which could have influenced our findings. Secondly, from an ethical viewpoint, we did not want to have fish with structural abnormalities to develop into adulthood with the potential suffering. Thirdly, the aim of our underlying hypothesis was to assess the more subtle effects of GC manipulation during early development possibly mimicking those that might occur in settings where polymorphisms of the PR receptor could influence their action on the developing heart. By adopting this novel experimental approach we observed adult hearts, derived from GR Mo treated embryos that were significantly smaller than controls with poorly developed trabecular network and reduced thickness of compact myocardium. The level of knockdown of Gr was around 30% at 120 hpf, by adulthood however, there was restoration of Gr protein levels to normal. This return of Gr expression by 120 dpf was clearly unable to restore normal cardiac structure and function. Indeed the histological appearance of GR Mo adult hearts was more similar to those of a normal juvenile heart at 40 dpf (Gupta et al., 2013).

The lack of maturity of GR Mo hearts is reflected in the lower expression of *vmhc* mRNA which also could have impacted on cardiac contractility. The increase in *plb* gene expression might also be predicted to reduce sarcoplasmic reticulum calcium uptake leading to altered cardiac diastolic function. However, the most striking difference in cardiac gene expression observed in these hearts was the reduction in *gata4* and an increase in *mef2c* abundance. Both of these genes are linked with early proliferative processes in the heart and the reduction in *gata4* could therefore account for the smaller number of cardiomyocytes detected in the GR Mo embryonic hearts. Increased *mef2c* may reflect the immature state of the hearts and could also reflect ongoing mechanisms attempting to compensate for reduced cardiac function. Taken together, this combination of altered structure and expression of important contractile and cardiac genes might be expected to impair cardiac performance as confirmed by echocardiographic Doppler indices demonstrating ventricular inflow signals consistent with increased ventricle stiffness.

The effects of GR Mo treatment may have been partly related to overall developmental delay. However, a direct impact on maturation and development of the cardiovascular system is also likely. Indeed, altered cardiomyocyte number and abnormal sarcomere patterning observed in GR Mo embryos, were rescued by *gr* mRNA lending support for a key role played by GCs in cardiomyocyte proliferation and function.

### 4.2 Increased glucocorticoid action during embryogenesis accelerates maturation of the embryonic heart

Exposure to excess synthetic corticosteroids during foetal life has been extensively studied in mammalian models including rats, mice and sheep (Fowden and Forhead, 2004; Nyirenda et al., 1998). These have been considered as classical models of foetal programming. There have been no similar studies using short-term exposure in zebrafish until now. One particular advantage of the fish is that there is very little maternal influence on corticosteroid metabolism during early embryonic development. This is important as maternal influences during pregnancy and lactation are typically confounding issues in experimental models where exogenous corticosteroids are administered to pregnant mammals.

While it is known that prolonged exposure to GCs in adult animals causes cardiac remodelling and dysfunction (Walker, 2007) there have been no studies exploring whether a brief exposure during embryonic life results in structural or functional changes in the foetus and whether the effects are carried into the adult. Previous studies injecting zebrafish embryos with cortisol did demonstrate a high proportion with significant cardiac structural defects (Nesan and Vijayan, 2012). In contrast, we saw few embryos with gross cardiac deformities despite using relatively high doses of Dex in the bathing water. Our study clearly shows that exposure of embryos to excess Dex led to a larger more mature embryonic heart with a higher expression of ventricular myosin heavy chain. These hearts also had fewer cardiomyocytes despite being larger suggesting cellular hypertrophy. This is plausible given that GCs have previously been shown to induce hypertrophy in cultured cardiomyocytes (Ohtani et al., 2009). In adults derived from these Dex-treated embryos, hearts were larger and heavier than controls. However, other classical histological features of cardiac hypertrophy typically seen in mammalian hypertrophy such as sarcomere reorganisation, altered vascular patterning and interstitial fibrosis were absent. Unfortunately, the zebrafish heart has a complex and irregular arrangement of cardiomyocytes such that we were unable to obtain a consistent cellular alignment in order to measure cardiomyocyte cross-sectional area in adult hearts. Therefore, we cannot fully exclude the possibility that the heart enlargement seen in adult Dex hearts was in fact due to an excess of non-cardiomyocyte cells or due to cardiomyocyte hyperplasia. However, given our findings in the embryo suggesting cardiomyocyte hypertrophy we can be more certain that this was also present in the adult zebrafish heart. In addition, we found increased levels of *vmhc* mRNA levels in adult Dex-hearts. Increased levels of *vmhc* mRNA (homologous to mammalian β-MHC) have been reported in a transgenic zebrafish model of cardiac hypertrophy (Chen et al., 2013) supporting the possibility that Dex adult hearts were enlarged due to cardiomyocyte hypertrophy.

Calcium handling genes were altered in adult Dex-hearts with reduced levels of *ryr* and increased levels of *plb* with no change in *serca*. This combination of changes in calcium cycling genes, if reflected in protein expression, would be likely to reduce calcium uptake and release possibly leading to altered contractile function. We were unable to detect changes in Doppler flow patterns in Dex-treated hearts and there were also no changes in ejection fraction. This could have been due to a lack of sensitivity of echocardiography to detect a change at the whole organ level.

Dex adult hearts demonstrated reduced levels of *mef2c* which is known to play an important role in cardiac development (Yu et al., 2013) and in inducing cardiomyocyte phenotype in cultured human fibroblasts (Wada et al., 2013). Previous reports also indicate that *mef2c* and *Gr* act cooperatively in controlling gene transcription, with GR influencing Mef2 activity (Speksnijder et al., 2012). The mef2 transcription factor family is important in the regulation of cardiac energy metabolism including cardiac fatty acid oxidation and maintenance of mitochondrial function (Czubryt and Olson, 2004). Low *mef2c* mRNA levels could therefore reflect an alteration in a number of cellular processes including proliferative capacity; control of transcription of GR related genes and cardiac metabolism.

Benato and colleagues (Benato et al., 2014), using a glucocorticoid-responsive zebrafish transgenic line found high expression of their transgene in the cardiac district of 2 dpf embryos and in the heart of 5 dpf larvae as well as in adult fish (particularly in the ventricular epicardium). This is very interesting given the subtle changes induced by embryonic Dex treatment in our adult hearts. Over-activation of GR in rodents has also been shown to alter adult cardiac physiology mainly mediated by conduction defects (Sainte-Marie et al., 2007). Again this suggests that GC signalling plays a key role in cardiomyocyte function and remodelling after insults in teleost fish, as shown in mice (Ren et al., 2012).

## 5 Study limitations

Much of the work presented in the current work has focused on GR-mediated actions of GCs. Since GR modulation was the aim of this study one may assume that alterations in cardiovascular structure and physiology are as a direct effect of GR-mediated actions. However, as the ligand for the mineralocorticoid receptor (MR), the other known receptor for corticosteroids, has not been identified in the zebrafish and as there appears to be limited *11* *βhsd2* mRNA found in the heart (Tokarz et al., 2013); it is likely that GC stimulates both MR and GR in the zebrafish heart (Cruz et al., 2013). Therefore, some of our observations, may have resulted from activation of MR. To understand the specific roles played by each of the receptors, targeted Mr manipulation could be carried out concurrently with the Gr modulation as previously reported in the mouse (Ren et al., 2012).

GCs are known to exert genomic effects by binding to, and activating, the transcription factors MR and GR. However, non-genomic effects have also been observed in mammals (Stellato, 2004) and more recently in zebrafish (De Marco et al., 2013). The mechanisms underlying the non-genomic effects are poorly understood and it is unclear whether non-genomic and genomic effects interact (De Marco et al., 2013). It is likely that Dex, particularly at the higher concentration used here [100 μM], is producing genomic and non-genomic effects since high concentrations are more likely to cause receptor saturation allowing free drug to have non-receptor mediated (non-genomic) effects. The steroid-binding characteristics of the receptors may also determine whether genomic or non-genomic effects are involved. A further interesting aspect, not reported here, is comparison of Mo treatment with treatment with the drug RU486 (a GR and progesterone receptor (PR) antagonist). We have previously used this drug in embryonic exposure studies (Wilson et al., 2013), and while many of the cardiovascular features observed were consistent with those observed in GR Mo treated embryos we also identified a number of non-specific effects which limit its use in this setting.

## 6 Future research

Future research could include the use of a number of recently published GR transgenic and mutant lines in similar experiments to those described here. A novel GR reporter line (Benato et al., 2014) could provide a way of assessing the impact of early life GC manipulation on expression of GR-related genes in a number of tissues throughout the embryos including the heart. Tracing these effects into adulthood would also be possible in this line. The Gr mutant zebrafish line (gr (s357)), where the transcriptional activity of GR is blocked (Griffiths et al., 2012; Muto et al., 2013) could also provide a novel approach to examining the impact of early perturbations in GR action on development and function of the cardiovascular system. This mutant line could also provide a method of dissecting the effects of maternal and endogenous GR transcription.

## 7 Conclusions

We have clearly shown that short term alterations in GC action during embryogenesis result in long-term alterations in structure, function and genetic composition of the heart. These changes result in measurable effects on cardiac contractile function. This could have important implications for human disease in that early life events could have subtle long term effects on the heart thus predisposing to altered response to injury or stress in adult life.

## Gene nomenclature used throughout will be as follows (unless highlighted in text)

Example, glucocorticoid receptor (GR).

*GR*, human (Homo sapiens) GR gene isoform, protein designation GR;

*Gr*, rodent (Mus musculus, Rattus norvegicus) GR gene isoform; protein designation GR;

*gr* zebrafish (*Danio rerio*) GR gene isoform, protein designation Gr.

## Funding statement

This study was funded by a British Heart Foundation PhD Fellowship award (KW) grant number RE/09/053. The work was also supported by the British Heart Foundation Centre of Research Excellence award (CoRE) grant number RE/08/001/23904.

## Conflicts of interest

No conflicts of interest. All authors takes responsibility for all aspects of the reliability and freedom from bias of the data presented and their discussed interpretation.

## Appendix A Supplementary data

**Control statistical comparison**. Statistical analysis of glucocorticoid receptor (GR) manipulation controls. Data has been represented graphically throughout as a mean of both dexamethasone (Dex) vehicle only control and GR morpholino (GR Mo) mismatch (mism) control. This table displays that no statistical differences were observed when comparing both control groups to each other.

**Gene primer sequences**. A table of the genes investigated by qRT-PCR with accession number, forward and reverse primer sequence. Genes investigated were Glucocorticoid receptor (*gr*), ventricular myosin heavy chain (*vmhc*), phospholamban (*plb*), myocyte enhancer factor 2 c (*mef2c*), GATA transcription factor 4 (*gata4*), Homeobox protein NKX2.5 (*nkx2.5*), Insulin like growth factor (*igf*), Ryanodine receptor (*ryr*), sarco-endoplasmic reticulum Ca2+ ATPase (*serca*), elongation factor 1 alpha (*ef1α*) and Glyceraldehyde 3-phosphate dehydrogenase (*gapdh*).
