# Supplementary material for: Early-life perturbations in glucocorticoid activity impacts on the structure, function and molecular composition of the adult zebrafish (Danio rerio) heart
Source: Mol Cell Endocrinol. 2015 Oct 15;414:120–31. doi: 10.1016/j.mce.2015.07.025 (PMC4562295; doi:10.1016/j.mce.2015.07.025)
Supplement: Supplementary file 1 [file mmc1.pptx]

## Slide 1
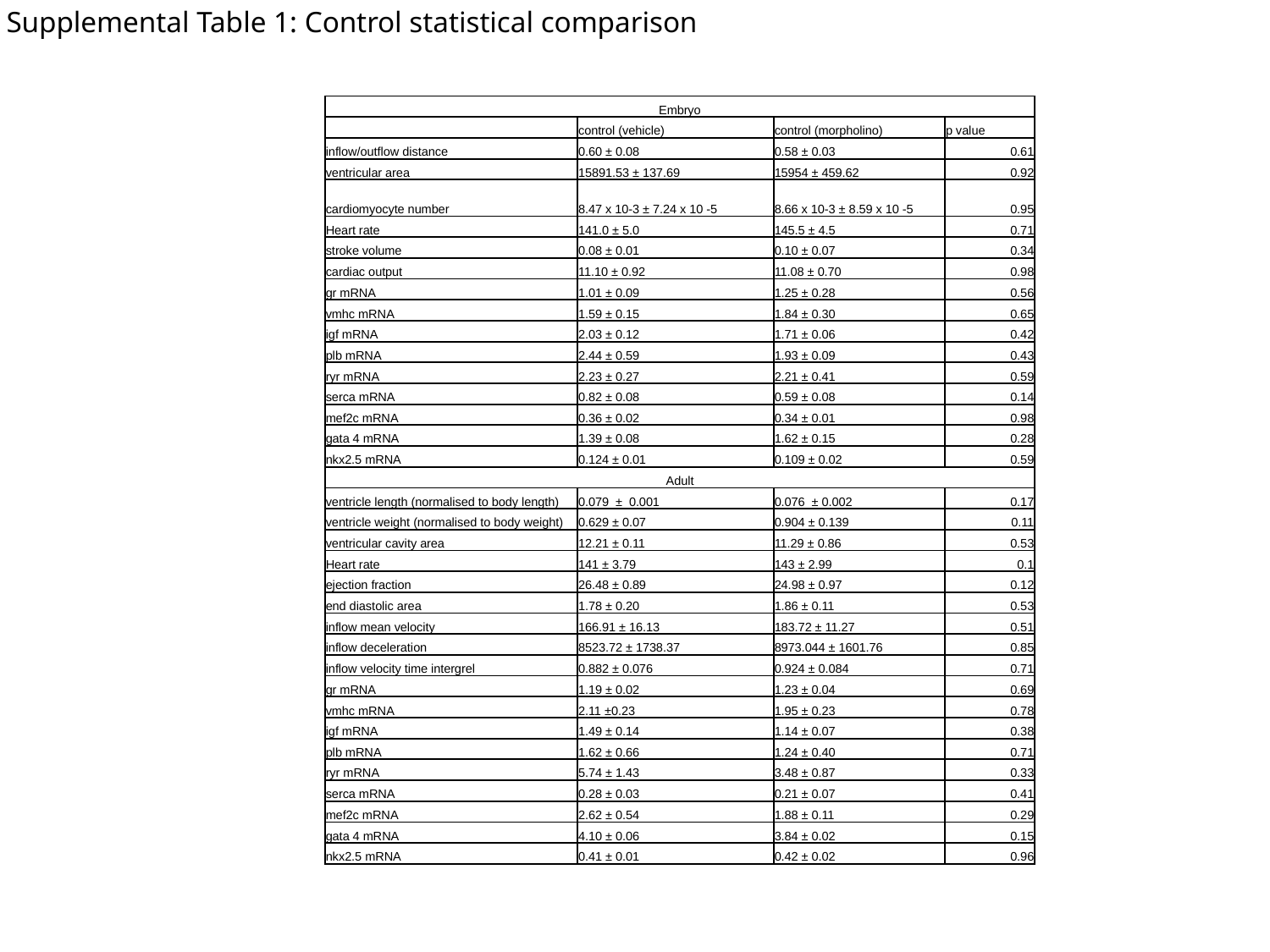

Supplemental Table 1: Control statistical comparison
| Embryo | | | |
| --- | --- | --- | --- |
| | control (vehicle) | control (morpholino) | p value |
| inflow/outflow distance | 0.60 ± 0.08 | 0.58 ± 0.03 | 0.61 |
| ventricular area | 15891.53 ± 137.69 | 15954 ± 459.62 | 0.92 |
| cardiomyocyte number | 8.47 x 10^-3^ ± 7.24 x 10 -5 | 8.66 x 10^-3^ ± 8.59 x 10 -5 | 0.95 |
| Heart rate | 141.0 ± 5.0 | 145.5 ± 4.5 | 0.71 |
| stroke volume | 0.08 ± 0.01 | 0.10 ± 0.07 | 0.34 |
| cardiac output | 11.10 ± 0.92 | 11.08 ± 0.70 | 0.98 |
| gr mRNA | 1.01 ± 0.09 | 1.25 ± 0.28 | 0.56 |
| vmhc mRNA | 1.59 ± 0.15 | 1.84 ± 0.30 | 0.65 |
| igf mRNA | 2.03 ± 0.12 | 1.71 ± 0.06 | 0.42 |
| plb mRNA | 2.44 ± 0.59 | 1.93 ± 0.09 | 0.43 |
| ryr mRNA | 2.23 ± 0.27 | 2.21 ± 0.41 | 0.59 |
| serca mRNA | 0.82 ± 0.08 | 0.59 ± 0.08 | 0.14 |
| mef2c mRNA | 0.36 ± 0.02 | 0.34 ± 0.01 | 0.98 |
| gata 4 mRNA | 1.39 ± 0.08 | 1.62 ± 0.15 | 0.28 |
| nkx2.5 mRNA | 0.124 ± 0.01 | 0.109 ± 0.02 | 0.59 |
| Adult | | | |
| ventricle length (normalised to body length) | 0.079 ± 0.001 | 0.076 ± 0.002 | 0.17 |
| ventricle weight (normalised to body weight) | 0.629 ± 0.07 | 0.904 ± 0.139 | 0.11 |
| ventricular cavity area | 12.21 ± 0.11 | 11.29 ± 0.86 | 0.53 |
| Heart rate | 141 ± 3.79 | 143 ± 2.99 | 0.1 |
| ejection fraction | 26.48 ± 0.89 | 24.98 ± 0.97 | 0.12 |
| end diastolic area | 1.78 ± 0.20 | 1.86 ± 0.11 | 0.53 |
| inflow mean velocity | 166.91 ± 16.13 | 183.72 ± 11.27 | 0.51 |
| inflow deceleration | 8523.72 ± 1738.37 | 8973.044 ± 1601.76 | 0.85 |
| inflow velocity time intergrel | 0.882 ± 0.076 | 0.924 ± 0.084 | 0.71 |
| gr mRNA | 1.19 ± 0.02 | 1.23 ± 0.04 | 0.69 |
| vmhc mRNA | 2.11 ±0.23 | 1.95 ± 0.23 | 0.78 |
| igf mRNA | 1.49 ± 0.14 | 1.14 ± 0.07 | 0.38 |
| plb mRNA | 1.62 ± 0.66 | 1.24 ± 0.40 | 0.71 |
| ryr mRNA | 5.74 ± 1.43 | 3.48 ± 0.87 | 0.33 |
| serca mRNA | 0.28 ± 0.03 | 0.21 ± 0.07 | 0.41 |
| mef2c mRNA | 2.62 ± 0.54 | 1.88 ± 0.11 | 0.29 |
| gata 4 mRNA | 4.10 ± 0.06 | 3.84 ± 0.02 | 0.15 |
| nkx2.5 mRNA | 0.41 ± 0.01 | 0.42 ± 0.02 | 0.96 |

## Slide 2
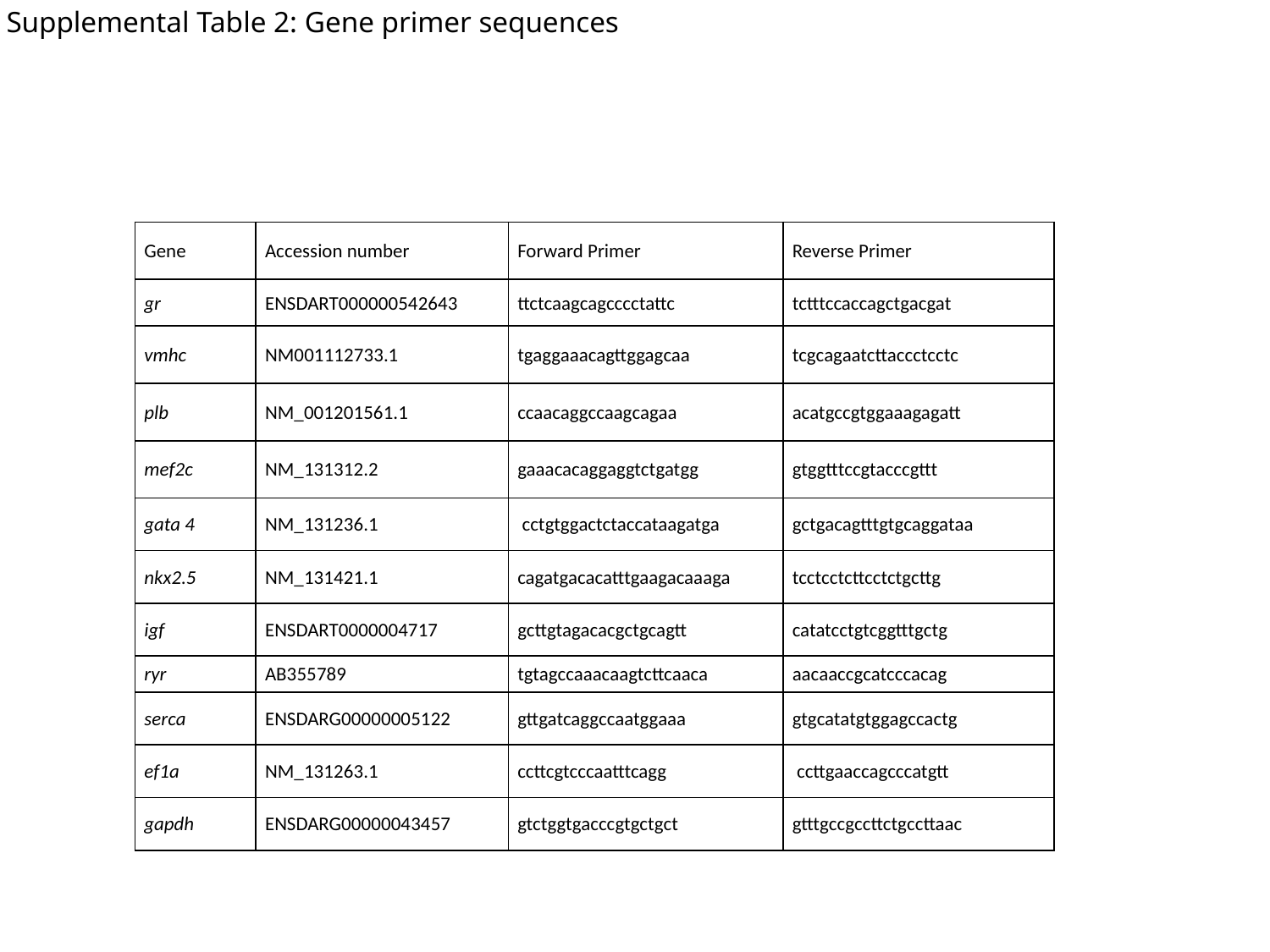

Supplemental Table 2: Gene primer sequences
| Gene | Accession number | Forward Primer | Reverse Primer |
| --- | --- | --- | --- |
| gr | ENSDART000000542643 | ttctcaagcagcccctattc | tctttccaccagctgacgat |
| vmhc | NM001112733.1 | tgaggaaacagttggagcaa | tcgcagaatcttaccctcctc |
| plb | NM\_001201561.1 | ccaacaggccaagcagaa | acatgccgtggaaagagatt |
| mef2c | NM\_131312.2 | gaaacacaggaggtctgatgg | gtggtttccgtacccgttt |
| gata 4 | NM\_131236.1 | cctgtggactctaccataagatga | gctgacagtttgtgcaggataa |
| nkx2.5 | NM\_131421.1 | cagatgacacatttgaagacaaaga | tcctcctcttcctctgcttg |
| igf | ENSDART0000004717 | gcttgtagacacgctgcagtt | catatcctgtcggtttgctg |
| ryr | AB355789 | tgtagccaaacaagtcttcaaca | aacaaccgcatcccacag |
| serca | ENSDARG00000005122 | gttgatcaggccaatggaaa | gtgcatatgtggagccactg |
| ef1a | NM\_131263.1 | ccttcgtcccaatttcagg | ccttgaaccagcccatgtt |
| gapdh | ENSDARG00000043457 | gtctggtgacccgtgctgct | gtttgccgccttctgccttaac |
